# A qualitative exploration of community perception on dementia and dementia care in Nepal

**DOI:** 10.1093/geroni/igaf122.4053

**Published:** 2025-12-31

**Authors:** Srijana Shrestha, Grace DiBacco, Jene Shrestha, Rekha Khatri, Bibhav Acharya, Sabitri Sapkota

**Affiliations:** Wheaton College, Norton, Massachusetts, United States; University at Albany (SUNY), Albany, New York, United States; Possible Nepal, Kathmandu, Bagmati, Nepal; University of Sydney, Sydney, New South Wales, Australia; University of California, San Francisco, San Francisco, California, United States; Possible Nepal, Kathmandu, Bagmati, Nepal

## Abstract

Around 2/3rd of those with dementia live in low- and middle-income countries (LMICs), which face the greatest burden due to limited financial resources and underdeveloped healthcare systems and weak social care networks. However, research on supporting people with dementia (PwD) in these settings is sparse. Our exploratory qualitative study examined dementia care through the perspectives of family caregivers, community health workers (CHWs), primary healthcare providers, and a key informant from the government in Nepal. We conducted focus groups with CHWs and individual in-depth interviews with other study participants. Interviews lasted 25-90 minutes and all audio tapes were transcribed and analyzed using thematic analysis. Study findings showed that the family caregivers and CHWs often attributed memory loss to normal aging, and weren’t aware of dementia as a health condition with available resources to help people with dementia and their caregivers but were eager to learn. All interviewees highlighted caregiver burden and the need for caregiver support, especially within the cultural context that expects family members to care for their seniors. Finally, study participants noted the need for institutional support noting the lack of dementia care training for health care providers, need for dementia care capacity building, and an urgent need for government programs and additional support for PwD and their caregivers. Finally, a unique perspective on cultural and community-based care was emphasized. Our study underscores several key challenges and necessary actions to improve dementia care in Nepal.

